# AI-assisted history education in China: a secondary evidence map of psychological antecedents and learning outcomes

**DOI:** 10.3389/fpsyg.2026.1956962

**Published:** 2026-09-23

**Authors:** Sicheng Jiang, Pei Wang, Yu Lu

**Affiliations:** 1 Simon Kuznets Kharkiv National University of Economics, Kharkiy, Ukraine; 2 School of Physics and Science and Technology, Hebei University, Baoding, Hebei, China; 3 School of Educational Science, Jiangsu Normal University, Xuzhou, Jiangsu, China

**Keywords:** AI-assisted history education, engagement, historical thinking, learning outcomes, motivation, psychological antecedents, secondary evidence mapping, self-efficacy

## Abstract

**Introduction:**

Evidence on the use of artificial intelligence in Chinese history education remains fragmented. This study mapped the available secondary evidence and contextualized it with targeted corroboration of history-specific studies.

**Methods:**

We analyzed a public bibliographic export of 1,063 records current to 1 April 2024 and conducted targeted full-text corroboration to identify history-education evidence beyond the original repository. After sequential screening, 30 records were retained for the contextual map. An expanded synonym audit was also performed to assess whether any direct history-education studies were missed.

**Results:**

Nine records explicitly identified mainland China, Hong Kong, or Taiwan as the study setting, including five primary empirical studies. The synonym audit did not identify a direct history-education study in the repository. Targeted corroboration identified one 2025 mainland-China study of AI-assisted secondary-school history teaching, two systematic reviews describing broader history-education literature, and one adjacent cultural-heritage education study. In the regional subset, emotion/anxiety/cognitive-load outcomes were reported in five records, engagement in four, motivation and self-efficacy in three each, skills/competence in six, and knowledge and performance in five each.

**Discussion:**

The repository should be interpreted as a contextual map of secondary evidence rather than a comprehensive estimate of the field. Overall, the evidence suggests emerging but still limited and heterogeneous attention to AI in Chinese history education.

## 1 Introduction

AI is now being applied in the field of education in various ways, including tutoring systems, automated feedback, learning analytics, educational robotics, and generative tools. In the history field, these systems can be used to help with source comparison, making timelines, creating questions, and providing feedback on writing with evidence. They also might make up citations, muddy up divergent interpretations, and make ambiguous claims with false confidence. The focus is thus on the circumstances in which AI can aid in disciplined historical inquiry, and the circumstances in which it can replace independent historical thinking. Although we still lack conclusive evidence, it suggests that selected learning activities can be facilitated with generative AI, but results depend on different kinds of tasks, student preparation, and instructional scaffolding (Ding et al., 2023; Escalante et al., 2023; Essel et al., 2024; Hwang et al., 2020; Zhai et al., 2021).

The Chinese context is particularly relevant due to China's vast educational digital infrastructure, its rapid adoption of AI technologies, and the significant regional, institutional, and learner heterogeneity. History is also a challenging subject in which to teach—pupils have to be able to identify evidence and make assertions, to situate claims within context, to compare points of view and draw conclusions from incomplete information. The practices are consistent with historical thinking practices that have been identified (Wineburg, 1991; Reisman, 2012; Seixas and Morton, 2013). These practices may be aided by AI but only when the output of the AI is used as a claim to test, not as a truth.

Intelligent tutoring, prediction, assessment, personalization, and generative AI have been reviewed and mapped throughout education previously. These reviews also identify learner agency, engagement, transparency, and teacher-mediated design as recurring concerns (Roll and Wylie, 2016; Zawacki-Richter et al., 2019; Chen et al., 2020; Zhai et al., 2021; Ouyang and Jiao, 2021; Chiu et al., 2023; Crompton and Burke, 2023; Ansari et al., 2024). However, they do not specifically examine AI-assisted history education in China. The lack of an obvious history-specific corpus is itself significant, since knowledge and source criticism play a major role in the safe use of generative systems.

A group of factors referred to as “psychological antecedents” can help to account for how the same AI tool can help one history learner and be confusing to another. Motivation is a factor in students' persevering with difficult sources. Whether or not they test an interpretation or the first fluent answer depends on self-efficacy. Attention is correlated to emotion and cognitive load when comparing and contextualizing. The trust in AI by learners impacts the likelihood of checking whether sources and claims are generated are verified independently. Once access, what happens is a function of engagement and self-regulation. These are located between the tool and the quality of historical reasoning (Ryan and Deci, 2020; Vansteenkiste et al., 2020; Chiu, 2021, 2022; Guay, 2022).

There are multiple learning outcomes in the field of history education. Immediate recall is helpful, but doesn't show historical thinking. Sourcing, corroboration, contextualization, chronological reasoning, perspective taking, and evidence-based argument are all forms of stronger evidence. The use of AI can enhance surface learning, but it has the potential of not reinforcing these deeper learning practices. It can also produce grammatically correct writing that hides poor arguments. Product measures, then, should be supplemented by process measures such as source checking, quality of explanations, prompt development, accuracy of citations, transfer to unfamiliar texts, and delayed retention (Wineburg, 1991; Reisman, 2012; McGrew et al., 2018).

In China, there have been significant developments in educational AI research related to self-regulation, academic outcomes, AI competence, and student attitudes. Publications issued since the repository cut-off are mentioned only as background for the repository and not counted in the repository. A study conducted in 2025 in the city of Guangzhou (Li and Liao, 2025) explored the application of AI teaching models in secondary school history classrooms by observing the classroom and analyzing it qualitatively from the teacher's perspective. This later evidence also indicates that if a direct history study is not found in the repository for 2024, it should not be taken as a sign that the field is not filled, but rather that this is a coverage limitation of that source. While other later research on the subject of self-efficacy, teacher care, and strategic applications of AI is not included within the mapped repository denominators, it serves as context (Fan et al., 2025; Jia et al., 2025; Liu et al., 2025).

This study is based on the systematic screening of a fixed public bibliographic export, which is complemented by a targeted history-education full-text check. Its contribution is the ability to differentiate between evidence of contextual information that can be found in the repository and directly relevant evidence of history-education information that can be found outside the repository. The evidence map thus records not only the evidence, but also the evidence that is missing from the supplied corpus and translates the patterns that repeatedly occur into testable propositions for the Chinese history classroom. The original export was done up to 1st April 2024; later exports for contextual or targeted corroboration are reported separately from the repository analysis.

## 2 Conceptual background

### 2.1 AI-assisted learning as a socio-technical process

The use of AI in education is frequently referred to by the functions it can perform: diagnosis, recommendation, tutoring, generation, prediction, and feedback. Although these functions are valuable, learning history is not simply a general information task. It is based on the quality, source, context and connection of sources. A system can offer quick assistance and limit interpretation or add unproven evidence. Thus, there is an interaction between the tool, the learner, the teacher, the task, the source set, and the institutional rules, which creates the value of the education (Chen et al., 2020; Khosravi et al., 2022).

The socio-technical approach is particularly relevant in the field of generative AI. A single system may serve as a tutor, editor, translator, planner, or answer generator. It can summarize documents, pose questions, or act as a representation of opposing points of view in a history classroom. That same flexibility can lead to shortcuts, quotes made up on the spot or to false balance. In recent scholarship, however, the question of adoption has been extended to the consideration of motivation, agency, quality control, integrity, and responsible use (among others: Grassini, 2023; Dempere et al., 2023; Imran and Almusharraf, 2023; Rahman and Watanobe, 2023; Tlili et al., 2023; Kasneci et al., 2023; Farrokhnia et al., 2024).

### 2.2 Psychological antecedents

Motivation and interest have been a concern of educational psychologists for a long time, as they influence attention, effort, strategy use, and receptiveness to feedback. While AI can be appealing for curiosity, it also can distract from thinking about the convenience or the speed. In the face of performance pressures, learners might opt not to invest in sustained learning. Self-determination theory argues for autonomous motivation, characterized by a sense of ownership and higher levels of involvement, in comparison to controlled motivation, which is stimulated by pressure, reward, or avoidance. The autonomy aspect of AI can be beneficial in the case of meaningful choices, but can be a hindrance when it starts to plan and make judgments for the learner (Ryan and Deci, 2020; Chiu, 2024).

Self-efficacy is a learner's confidence in his/her competence in doing a task. It can dictate the usage of AI. Learners who have a high level of confidence can experiment with their ideas, compare explanations, and ask for feedback, while learners with a low level of confidence can take the output of the system for granted and without critical processing. Confidence is not always protective as it can decrease verification behavior. Instead, “calibrated confidence” is a more beneficial goal: They should know what they can do, what the system can do, and where it and they can fail (Delcker et al., 2024; Liu et al., 2025).

Emotion and cognitive load also influence the quality of learner-AI interaction. Anxiety may limit the evaluation resources, enjoyment may keep people exploring, and moderate frustration could mean they are engaging in a productive challenge. AI can de-load by structuring information, but it can also load when learners have to evaluate the correctness of the information, evaluate and compare alternative suggestions, or solve contradicting information. The effects are contingent on the experience of learners and the complexity of the task and should not be considered as one positive or negative effect.

The reason for aligning learning to the use of an AI system is not enough; attitude, perceived usefulness, acceptance, and trust are important factors that explain that learners will use it or not. It is possible to have an easy-to-use and broadly accepted tool that can facilitate shallow learning. So, there is a need to calibrate trust: not enough trust might stifle constructive interaction, and too much trust may result in acceptant participation. Whereas, in educational context, a balanced level of trust is more beneficial than high trust (Almogren et al., 2024; Chan and Hu, 2023).

Engagement and self-regulation are the mediators between psychological antecedents and learning outcomes. Engagement embraces behavioral, emotional, and cognitive commitment, and self-regulation involves planning, monitoring, adjusting plans, and reflecting. AI can assist with these processes by reminding, hinting, and giving feedback, and can also replace them by making decisions for the learner. The main problem of the system is either increasing or replacing learners' control over the system (Lo et al., 2024; Darvishi et al., 2024).

### 2.3 Learning outcomes

Historical thinking extends beyond achievement and performance, although factual recall remains an important component of history learning. Students should know the author and purpose of a source, the context in which the source was created, and how it relates to other evidence. Other aspects of historical reasoning are chronology, continuity, and change, causation, significance, and evidence-based argument. These outcomes should not be lumped together in a global statement of effectiveness (Wineburg, 1991; Seixas and Morton, 2013).

The use of generative AI in history education is particularly informative when both product and process measures are assessed. AI-generated responses may include invented quotations, incorrect chronology, or claims stated with greater certainty than the available evidence supports. Evaluation should therefore assess both the final product and the process through which it was produced, including how learners used sources, selected and cited evidence, developed prompts, revised interpretations over time, and defended their conclusions without AI assistance. The findings from other education fields suggest that benefits are more convincing with the use of pre-defined prompts, feedback, and teacher support (Deng et al., 2025).

The side effects are also significant, negative or unwanted. They run the risk of: cognitive offloading, dependency, lack of agency, privacy risk, bias, lack of equal access, and academic misconduct. It is fitting that these have been identified as “policy concerns” (Cotton et al., 2024; UNESCO, 2023). That does not, however, mean that the use of risk language is an alternative to measurement. Research should therefore adopt clear definitions and thresholds that discriminate between routine and problematic use of AI and, importantly, between learner assistance and learner reasoning being replaced by AI.

### 2.4 History education as a domain-specific case

AI use in history education requires particular care because historical claims are provisional, evidence-based, and open to interpretation. Students must learn to source documents, corroborate accounts, contextualize evidence, and explain how an argument was constructed. These activities differ from simple factual retrieval because they require judgment about provenance, omission, bias, and competing perspectives (Wineburg, 1991; Reisman, 2012).

A history-education evidence map must therefore identify the subject area explicitly and distinguish history-specific outcomes from general measures such as satisfaction, speed, or performance. The original repository was audited with dedicated history-domain terms and did not contain a directly identifiable history-education study. A later targeted full-text check identified sparse direct evidence outside the repository, including a mainland-China secondary-school history study (Li and Liao, 2025). The evidence gap is therefore one of limited and uneven coverage rather than complete absence.

### 2.5 Review questions

The review was guided by four questions:

- RQ1. What direct evidence on AI-assisted history education in China is visible in the original repository and in the targeted full-text corroboration set?
- RQ2. Which psychological antecedents and learning outcomes appear in the confirmed regional evidence, and which of these can be linked directly vs. only provisionally to history education?
- RQ3. How does the evidence differ by study type, educational sector, AI modality, and regional context?
- RQ4. What hypothesis-generating framework can guide direct empirical research on AI-assisted history education in China?

## 3 Methods

### 3.1 Secondary evidence-map design and reporting approach

This study used a secondary systematic evidence-mapping design applied to a fixed public bibliographic export. Evidence mapping was selected because the available material was heterogeneous in study design and outcome reporting, comparable effect estimates were unavailable, and the primary objective was to characterize evidence coverage and gaps rather than estimate a pooled intervention effect (James et al., 2016). PRISMA 2020 and PRISMA-ScR were used as reporting references for transparent identification, screening, and evidence charting (Page et al., 2021; Tricco et al., 2018). The study did not conduct a *de novo* multi-database search and should not be interpreted as a comprehensive systematic review of all Chinese or international literature. The protocol was not registered in advance.

The export did not include publication year, article title, journal, document type, or DOI fields for the candidate records. The repository was current to 1 April 2024. Records without a defensible date signal were not used for temporal claims, and no annual trend or year-based subgroup analysis was produced. Publications appearing after the repository cutoff were handled only in the separate targeted corroboration step or as contextual references.

### 3.2 Data source

The main data source was the public Bibliometric Dataset on AI in Education (Hanid, 2025) which is available on Mendeley Data, Version 1, DOI 10.17632/46xbj75f6n.1. The available export was current up to 1 April 2024 and consisted of 1,063 rows and 7 fields (author names, affiliations, author-affiliation strings, abstract, author keywords, index keywords, and EID). The repository is open, reproducible, citable, and versioned. It was used as a secondary bibliographic database, not as a complete database or full-text search.

An abstract field was available for all 1,063 records, although some abstracts represented summaries of conference proceedings. No missing text was imputed, and missingness was present in the affiliation and keyword fields. Regional classification required an explicit study-location signal in the abstract, keywords, or available bibliographic information. Author affiliation alone was not treated as evidence that data collection occurred in the corresponding region. Records with unresolved study locations were retained in the broad contextual map but excluded from regional thematic counts.

#### 3.2.1 Targeted post-corpus full-text corroboration

Four full-text reports supplied separately from the repository were inspected to test the interpretation of the original history-domain result. The corroboration set comprised a published 2025 mainland-China study of AI-assisted teaching in secondary-school history classrooms (Li and Liao, 2025); a 2026 systematic review of AI in history learning (Mahmudi et al., 2026); an undated manuscript supplied in full text that synthesizes 18 empirical studies in history education (Li et al., 2025); and a 2026 China-focused study of AI-assisted cultural-heritage education (Yu, 2026), which was treated as adjacent rather than direct school-history evidence. These reports were used for corroboration, citation discovery, and interpretation only; they were not added to the repository frequency denominators because they were obtained through a different pathway.

### 3.3 Eligibility criteria

Eligibility was operationalized using a Population-Concept-Context (PCC) structure suited to evidence mapping. Population covered learners, teachers, and educational participants; Concept covered educational AI use together with psychological constructs and learning outcomes; Context treated mainland China as the primary national setting while Hong Kong and Taiwan were recorded as separate regional strata. Evidence type and explicit history-domain relevance were coded as additional classification fields.

Table 1 summarizes the screening criteria that guided inclusion and exclusion at each stage of the review.

**Table 1:** Eligibility and classification rules used in the post-screening audit.

Domain	Inclusion rule	Exclusion rule
Population/context	Learners, teachers, or educational settings. Mainland China was the primary national context. Hong Kong and Taiwan were retained as separate regional strata when the study location was explicit. Direct history inclusion required a clear history-education or historical-thinking signal.	Affiliation alone was insufficient for regional classification. Location-unresolved records were not used for region-specific counts. A study explicitly located in South Korea was retained only in the broad contextual map and excluded from the regional analysis.
Intervention/exposure	AI used for teaching, learning, assessment, feedback, prediction, tutoring, robotics, analytics, or generative support.	AI mentioned without an educational function.
Psychological construct	At least one clearly identifiable learner or teacher construct, such as motivation, emotion, cognitive load, engagement, self-efficacy, attitude, trust, agency, or self-regulation.	Construct absent or coded only as other/unclear.
Learning outcome	At least one clearly identifiable educational outcome, such as knowledge, skill, performance, critical thinking, efficiency, satisfaction, creativity, or retention.	Outcome absent or coded only as other/unclear.
Record type	Primary empirical studies were used for learning-effect interpretation. Reviews, proceedings, conceptual papers, and unclear records were retained only for evidence mapping.	Non-empirical records were not treated as proof of learning effects.
History-education relevance	Direct terms included history education, history teaching, history learning, historical thinking, historical reasoning, historical inquiry, historical literacy, history classroom, history curriculum, document-based inquiry, historical sources, sourcing, corroboration, contextualization, historiography, and related history-domain terms.	Broader terms such as social studies and humanities triggered manual checking but were not treated as direct history evidence without a clear history-specific context.
Corpus boundary	The supplied export was current to 1 April 2024; only records available within that corpus were screened.	No annual or year-specific claims were made for records without a defensible date signal.

### 3.4 Screening procedure

The repository screening was completed in three stages. First, predefined text rules identified records with an AI-in-education signal, a China text or affiliation signal, a psychological construct, and a learning outcome, producing 35 candidate records. Second, a record-level audit checked construct eligibility, outcome eligibility, evidence type, and study location. Five records were excluded because the antecedent or outcome remained other/unclear. The South Korea study was retained in the broad contextual map but excluded from the regional analysis, leaving 30 contextual records: nine with an explicit mainland China, Hong Kong, or Taiwan study signal, 20 location-unresolved records, and one South Korea record. Third, a history-domain audit searched abstracts and keywords for direct history-education terms. No direct history-education record was identified within the original repository.

Figure 1 presents the PRISMA 2020-adapted flow diagram for the secondary repository screening and record-level audit. A sensitivity audit then broadened the terminology to include machine learning, large language models, generative AI, ChatGPT, intelligent tutoring, social studies, humanities, historical inquiry, historical literacy, source evaluation, sourcing, corroboration, contextualization, chronology, and related terms. The expanded AI dictionary reproduced the same AI-screen count (*n* = 1,026). Thirteen records matched at least one broader history-adjacent term, but manual inspection did not identify a direct history-education study. This sensitivity check reduces, but does not eliminate, the possibility of false negatives caused by terminology variation.

**Figure 1:**
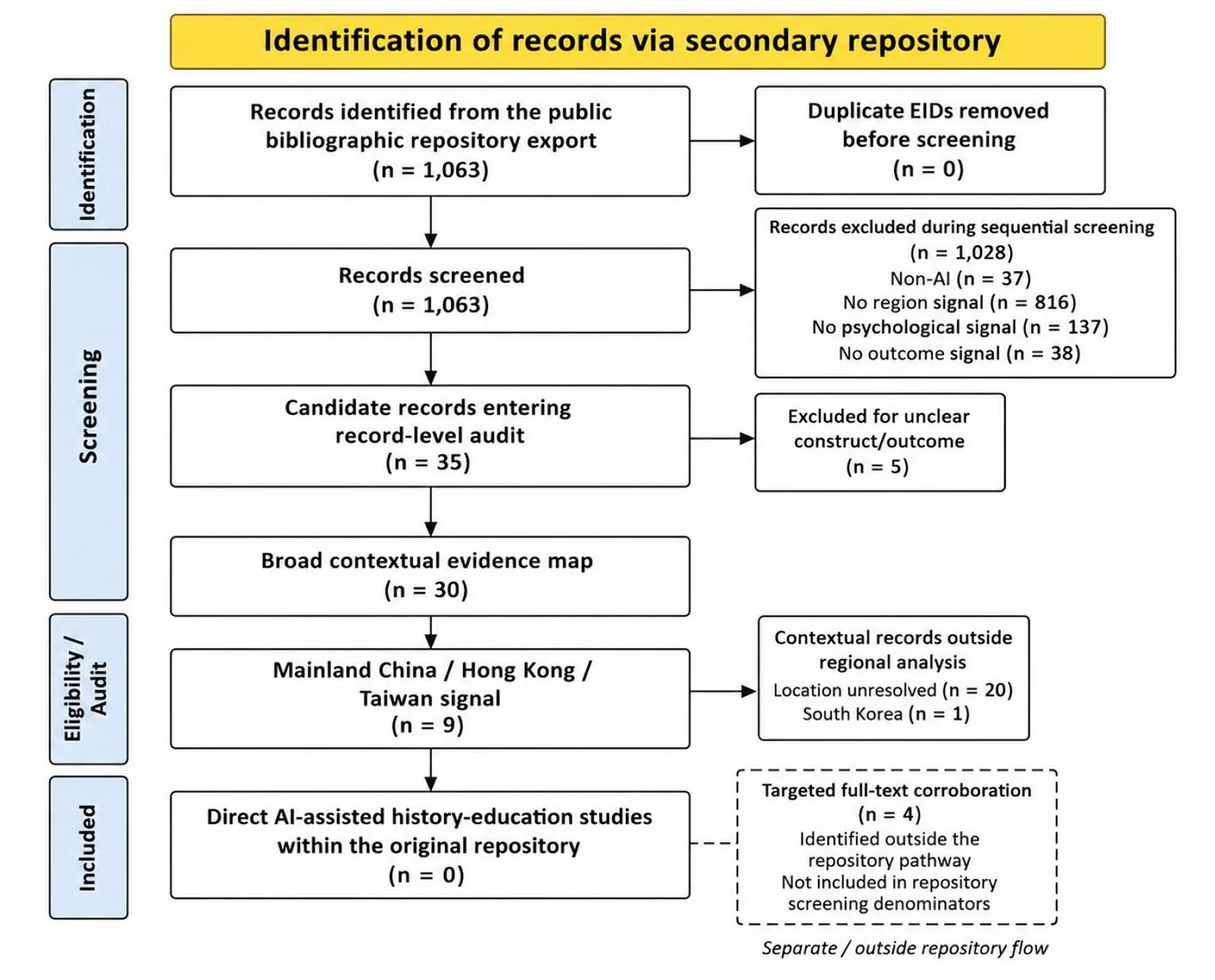
PRISMA 2020-adapted flow diagram for secondary repository screening and record-level audit. The four targeted full-text corroboration reports were identified outside the repository pathway and were therefore not included in the repository screening denominators.

### 3.5 Coding framework

Psychological constructs were coded into motivation or interest; emotion, anxiety, or cognitive load; engagement; self-efficacy, confidence, or competence; satisfaction or enjoyment; acceptance, attitude, or intention; self-regulation, agency, or autonomy; and trust or credibility. Learning outcomes were coded as knowledge/understanding; skills/competence; achievement/performance; problem solving/critical thinking; efficiency/productivity; satisfaction; creativity; and retention/persistence. Multiple codes could be assigned to a record, and co-occurrence was not treated as a tested statistical relationship. History-specific outcomes included sourcing, corroboration, contextualization, chronological reasoning, historical empathy, and evidence-based argument where explicitly reported.

Initial dictionary coding was followed by a second-pass record audit against the original abstract and keyword fields. The source archive did not contain an independent second-coder record, so inter-rater reliability cannot be reported. In addition, the first-pass category combining emotion, anxiety, and cognitive load was retained for continuity with the original counts even though these are conceptually distinct constructs; the combined frequency is therefore interpreted cautiously rather than as a single psychological mechanism.

The AI modality codes included: generative AI or chatbots, adaptive or intelligent tutoring systems, learning analytics or prediction, educational robotics, automated assessment or feedback, and general AI. The five types of evidence were coded on a separate basis: primary empirical study, review/synthesis, proceedings aggregate, conceptual or system design, and unclear. When evaluating learning-effect claims, reviews and proceedings were not included in the pool of primary studies.

### 3.6 Synthesis and confidence assessment

Descriptive counts and a critical comparative narrative were used. Due to the lack of effect sizes, variance estimates, intervention length, and similar outcome measures, an attempt to conduct a meta-analysis was not made. The 30-record contextual map specifies the extent for the provided repository (Table 2). Observed outcomes are limited to the five major empirical studies in the nine studies with an explicit mainland China or Hong Kong or Taiwan study signal, and statements regarding interpretation are limited to those specific to the region. The denominators are not combined with the targeted full-text reports, which are discussed separately.

**Table 2:** Repository screening and record-level audit counts.

Screening or audit stage	*n*	% of original
Records in supplied repository	1,063	100.0
AI-in-education signal	1,026	96.5
China-connected text or affiliation signal	210	19.8
Psychological-construct signal	73	6.9
Candidate records with learning-outcome signal	35	3.3
Excluded after construct/outcome audit	5	0.5
Retained broad contextual evidence map	30	2.8
Confirmed China/Hong Kong/Taiwan subset	9	0.8
Location-unresolved contextual records	20	1.9
External-location contextual record (South Korea)	1	0.1

Confidence was taken as a multi-layered concept and not a formal certainty-grading system. The broad map includes statements on the coverage of the repository; the nine-record regional subset includes cautious contextual observations; the five primary regional empirical studies include limited statements about the outcomes observed; and the targeted full-text corroboration includes direct history-education context outside of the repository. There were five main regional empirical studies, two of which were from Taiwan, two from Hong Kong and one from mainland China. The findings are not considered to be from a homogeneous educational context because there are differences in the curriculum, policy, language practices, and patterns of educational technology use across the settings.

### 3.7 Full-text status, classification limits, and quality-assessment boundary

The 35 repository candidates were verified at the level of the record by using the abstract, the keywords, the affiliation field, and the EID. The classification of evidence types and study design, therefore, may be considered preliminary and based on abstract information. The full-text retrieval may have the potential to change classifications of records that have less methodological detail. The post-corpus reports were targeted and were available in full text but were not captured from the repository pathway and were not used to retroactively add quality scores to the candidate set.

A complete report together with all relevant design-specific information (Hong et al., 2018) is a prerequisite for a defensible MMAT 2018 assessment. There were no study-level MMAT results or overall study-level quality scores for full reports of the original candidate set available. The MMAT readiness table provided as a supplement indicates what information would be needed for full-text appraisal, but is not to be used as a substitute for a quality assessment. This is considered a central limitation, since abstracts don't provide confidence about sampling, attrition, validity of the measurement, control of confounding, or fidelity of the intervention. The background, methodological and targeted corroboration sources listed in the reference list are not identical to the original candidate EIDs. For a few repository candidates, report-level titles and journals, years and DOI links could not be found, and are stated in the Supplementary material, not deduced.

### 3.8 PRISMA and PRISMA-ScR reporting status

The secondary evidence-map process was reported using PRISMA 2020 and PRISMA-ScR. Items that are applicable are classified as complete, partial, not applicable to the secondary repository design, or not completed in Supplementary Table S1. The reporting documents repository identification, screening rules, data-charting fields, descriptive synthesis, and the layered approach to confidence interpretation. A *de novo* multi-database search, full retrieval of all reports for the original candidate set, and full-text risk-of-bias assessment were not performed; these limitations are explicitly reported. The candidate audit, exclusion log, MMAT readiness table, regional study characteristics, screening dictionary, DOI audit, expanded terminology sensitivity audit, and targeted full-text corroboration log are also included in the Supplementary package.

## 4 Results

### 4.1 Screening results

The repository contained 1,063 records. The AI-in-education screen retained 1,026 records, the China text or affiliation screen retained 210, the psychological screen retained 73, and the learning-outcome screen produced 35 candidates. Five candidates were excluded after construct and outcome checking. The South Korea study was retained as contextual evidence but excluded from the regional analysis. Thirty records therefore remained in the broad contextual map, representing 2.8% of the repository. The history-domain audit identified no directly eligible history-education study within the original repository.

Nine records clearly specified mainland China, Hong Kong, or Taiwan as the study context. Twenty records remained location-unresolved, and one empirical record was explicitly located in South Korea. The nine confirmed regional records alone were used for regional theme counts; observed-outcome interpretation was further restricted to the five primary empirical studies. The separate targeted full-text corroboration identified direct history-education evidence outside the repository and is reported in Section 4.3.

### 4.2 Evidence profile

After the audit, the 30-record contextual map contained 17 primary empirical studies, three reviews or syntheses, three proceedings aggregates, one conceptual or system-design paper, and six records with unclear design reporting. These groups were analyzed separately. Observed learning-effect statements were drawn only from primary studies, while the direct history-education interpretation was kept separate from the broader contextual map. Method reporting remained uneven. Of the nine confirmed regional records, five were primary empirical studies, three were proceedings aggregates, and one was classified as unclear/other. The five empirical studies comprised two Taiwan studies, two Hong Kong studies, and one mainland-China study. Four abstracts reported samples of 24, 47, 12, and 750 participants; one case study did not state its sample. None of these five repository studies was identified as a history-classroom investigation. Table 3 summarizes the regional empirical records, while Supplementary Table S10 provides the available bibliographic details and stable identifiers.

**Table 3:** Confirmed regional primary empirical records identified from the supplied abstracts and linked by scopus EID.

Verified record identity	Context and sample	Design and system	Main abstract-level result
Chen et al. (2021) EID 2-s2.0-85110439050	Taiwan; 24 fourth-grade pupils	Classroom experiment; game-based intelligent robot	Lower cognitive load and higher efficiency were reported for boys; the performance difference was not significant.
Jeng and Chen (2022) EID 2-s2.0-85146954308	Taiwan; 47 pupils aged 10–11	Mixed-method comparison; digital Pentomino game with learning analytics	Experts and novices differed in spatial tests, operations, and problem-solving sequences.
Kohnke et al. (2023) EID 2-s2.0-85165243775	Hong Kong; 12 instructors	Qualitative interviews; generative AI for language teaching	Familiarity, confidence, institutional support, and professional-development needs were reported.
Cheng and Wang (2023) EID 2-s2.0-85178289347	Hong Kong; sample not stated	Qualitative case study; adaptive learning platform	Leadership support and student-centered pedagogy were reported alongside engagement and learning-quality claims.
Sun and Song (2023) EID 2-s2.0-85180606431	China; 750 vocational-college teachers	Cross-sectional survey; AI and big-data analytics	AI adoption and data accuracy were reported alongside teacher performance; the design does not establish causality.

The abstracts were sufficient to identify study design and sample information in several cases, but not to judge all MMAT criteria. Sampling strategy, attrition, measurement validity, confounding control, and intervention fidelity require the full report. Table 3 uses the verified author strings and Scopus EIDs from the supplied export; Supplementary Table S10 provides the available year and publisher signals together with stable Scopus record links.

Figure 2 summarizes the evidence-status distribution of the 30 records retained in the contextual evidence map.

**Figure 2:**
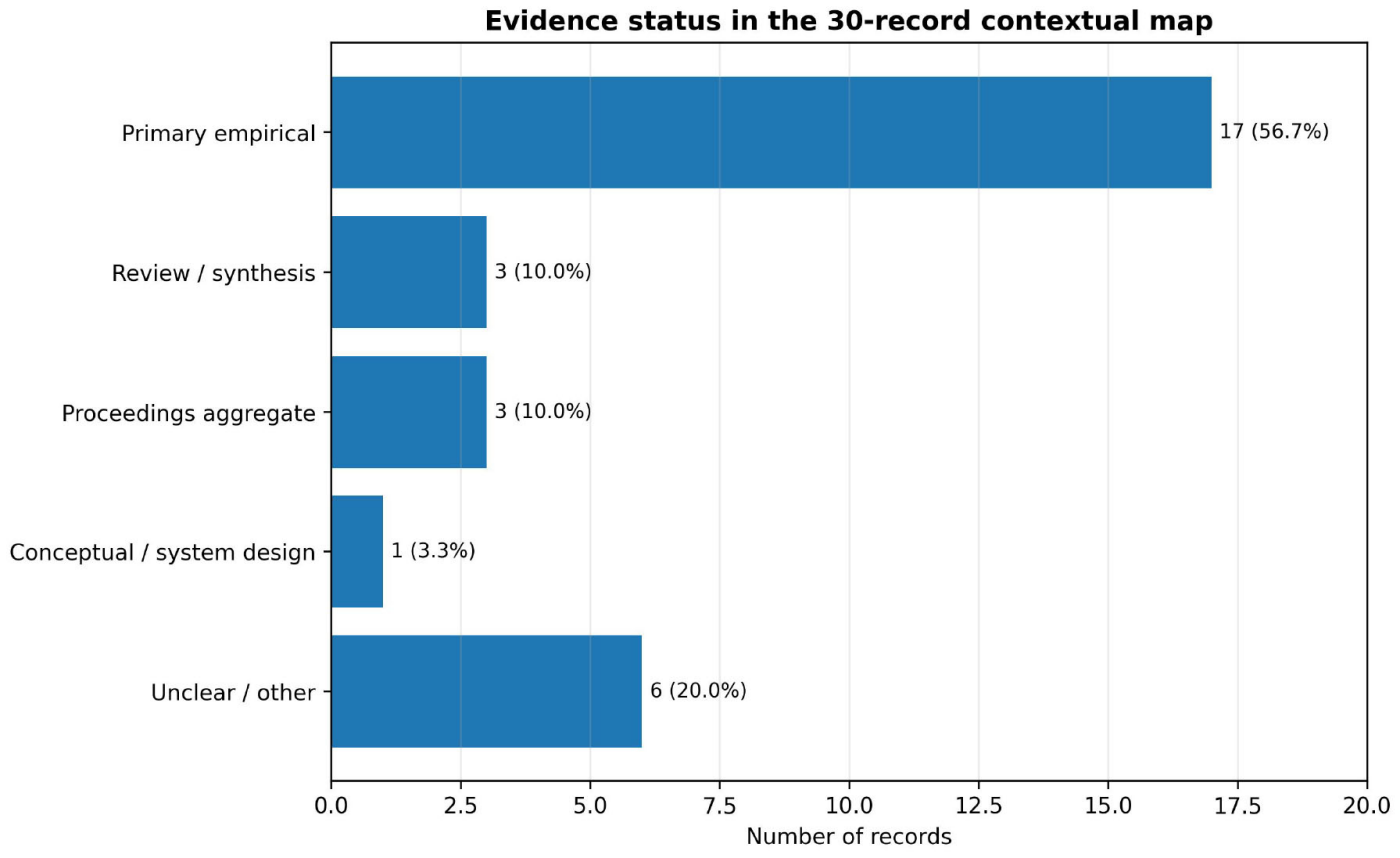
Evidence-status classification of the 30-record contextual map.

Figure 3 shows the distribution of educational settings represented among the 30 retained contextual records.

**Figure 3:**
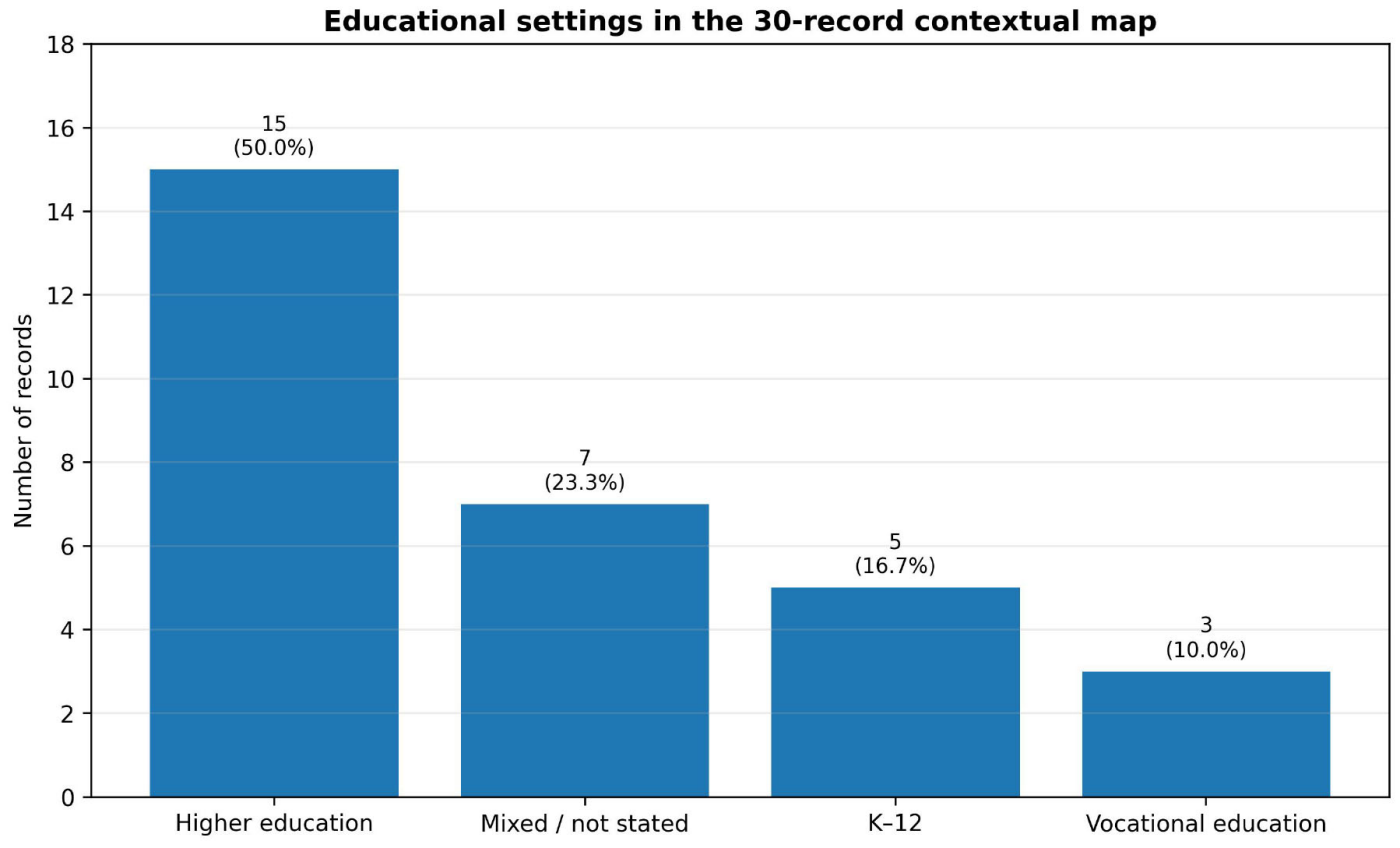
Educational settings represented among the 30 retained contextual records.

### 4.3 History-education evidence and targeted full-text corroboration

The original repository audit found no abstract or keyword containing a direct history-education signal, even after the broader terminology sensitivity check. This result is specific to the supplied repository and should not be interpreted as evidence that AI-assisted history education is absent from the wider literature. Targeted full-text corroboration changed that interpretation. The corroboration set included the published 2025 mainland-China classroom study by Li and Liao (2025), the 2026 systematic review by Mahmudi et al. (2026), an undated supplied manuscript review by Li et al. (2025), and the 2026 adjacent cultural-heritage education study by Yu (2026). Li and Liao (2025) report a directly relevant study of AI-assisted teaching in secondary-school history classrooms in Guangzhou, based on classroom observations and 36 history teachers from eight secondary schools. The two review sources indicate a wider history-education literature, whereas Yu (2026) provides adjacent China-focused evidence rather than direct school-history evidence. These sources show that direct history-education research exists beyond the original repository, but China-specific empirical evidence remains sparse and methodologically heterogeneous.

### 4.4 Psychological antecedents

Within the nine confirmed regional records, emotion, anxiety, or cognitive-load signals were identified in five records, engagement, or participation in four, motivation, or interest in three, and self-efficacy, confidence, or competence in three. Acceptance, attitude, or intention appeared in two records, while self-regulation, agency, or autonomy appeared in one. Satisfaction or enjoyment and trust or credibility were not clearly coded. Given the small denominator (*n* = 9), these results are reported as raw counts rather than percentages and should be interpreted as descriptive evidence-map frequencies rather than prevalence estimates.

Table 4 reports the confirmed-subset counts. The most informative regional material concerned cognitive load, attitudes toward AI, engagement, and motivation under structured educational conditions. Because the first-pass coding combined emotion, anxiety, and cognitive load, this category is interpreted as a broad signal rather than a single psychological mechanism. Direct history-classroom testing of these constructs remains limited.

**Table 4:** Psychological themes reported in the confirmed regional subset.

Psychological theme	*n*
Emotion, anxiety, or cognitive load	5
Engagement or participation	4
Motivation or interest	3
Self-efficacy, confidence, or competence	3
Acceptance, attitude, or intention	2
Self-regulation, agency, or autonomy	1
Satisfaction or enjoyment	0
Trust or credibility	0

Counts are based on the nine confirmed regional records. Multiple themes could be coded for a single record; therefore, counts are not mutually exclusive.

### 4.5 Learning outcomes

Within the nine confirmed regional records, skills or competence was identified in six records, knowledge, or understanding in five, achievement, or performance in five, problem solving, or critical thinking in four, and efficiency, or productivity in two. Learning satisfaction, creativity, and retention, or persistence were not clearly coded. Given the small denominator (*n* = 9), these results are reported as raw counts rather than percentages and should not be interpreted as precise prevalence estimates.

Confirmed-subset frequencies are given in Table 5. With respect to the five regional empirical studies, there is the strongest evidence related to immediate performance/efficiency, attitudes, engagement, and teacher outcomes. The targeted history-education reports provide direct disciplinary context, but are too varied to be summarized with pooled effect estimation.

**Table 5:** Learning-outcome themes reported in the confirmed regional subset.

Learning-outcome theme	*n*
Skills or competence	6
Knowledge or understanding	5
Achievement or performance	5
Problem solving or critical thinking	4
Efficiency or productivity	2
Learning satisfaction	0
Creativity	0
Retention or persistence	0

Counts are based on the nine confirmed regional records. Multiple learning-outcome themes could be coded for a single record; therefore, counts are not mutually exclusive.

### 4.6 AI modalities and direction of findings

The 30-record contextual map had 10 records with a Generative AI/Chatbot code, nine records with a Learning Analytics/Prediction code, eight records with an Adaptive/Intelligent Tutoring code, eight records with a General AI code, four records with an Education Robotics code, and one record with an Automated Feedback/Assessment code; some records were coded for more than one modality. Of the nine confirmed regional records, five were adaptive or tutoring systems, three were generative, three were educational robotics, two were analytics and one was general. No abstract reporting was used as an effectiveness rate. In positive terms, this might be a result of a selective focus on the benefits or positive systems rather than an actual measure of success. Therefore, the meaning of the outcome claims was understood in relation to the type of study, comparison condition, characteristics of the samples and task design.

A structured system was more likely to be used in a bounded task with explicit feedback rules, while a generative system was more likely to be used with less controlled activities. This difference may account for some of the concrete task outcomes reported for tutoring, compared with the outcomes reported in robotics studies. It does not indicate that one modality is superior in all cases. Structured tools can be used in history education to facilitate chronology or comparisons of sources, but they demand higher levels of verification for citation, source and interpretation. Table 6 is a summary of the education uses and interpretive limitations of each modality.

**Table 6:** AI modalities represented in the evidence map and their interpretive limits.

AI modality and map count	Educational role and interpretive limit
Generative AI or chatbot (30-map *n* = 10; confirmed *n* = 3)	Rapid dialogue, drafting, explanation, and feedback. Flexible use creates risks of substitution, hallucination, and weak source checking.
Adaptive or intelligent tutoring (*n* = 8; confirmed *n* = 5)	Personalized hints, sequencing, and practice. Strong task alignment may improve clarity but can narrow learning paths.
Learning analytics or prediction (*n* = 9; confirmed *n* = 2)	Risk detection, recommendation, and performance modeling. May target support but can reproduce bias or obscure reasoning.
Educational robotics (*n* = 4; confirmed *n* = 3)	Embodied, game-based, or social learning. May raise interest while adding cognitive load or access barriers.
Automated feedback or assessment (*n* = 1; confirmed *n* = 0)	Immediate evaluation and guidance. Quality and transparency require checking.
General AI (*n* = 8; confirmed *n* = 1)	Broad support across tasks. Claims are difficult to interpret without a defined tool and task.

Higher education had the highest diversity of uses and bounded, teacher-directed uses occurred more often in the K−12 and vocational settings. Emotion, engagement, motivation, and self-efficacy were each identified in multiple records, but the available evidence does not establish that any one of these factors is more influential than the others. Mediation, moderation, and comparative-effect hypotheses require direct empirical testing in full-text studies conducted specifically within history-education settings.

### 4.7 Hypothesis-generating history-education pathway

The conceptual pathway in Figure 4 organizes the evidence into four stages: psychological antecedents, learner-AI engagement, historical inquiry processes, and history-learning outcomes. Instructional design and governance are treated as contextual moderators. The pathway is used to structure coding and future research questions; it is not presented as a causal model estimated from the repository data.

**Figure 4:**
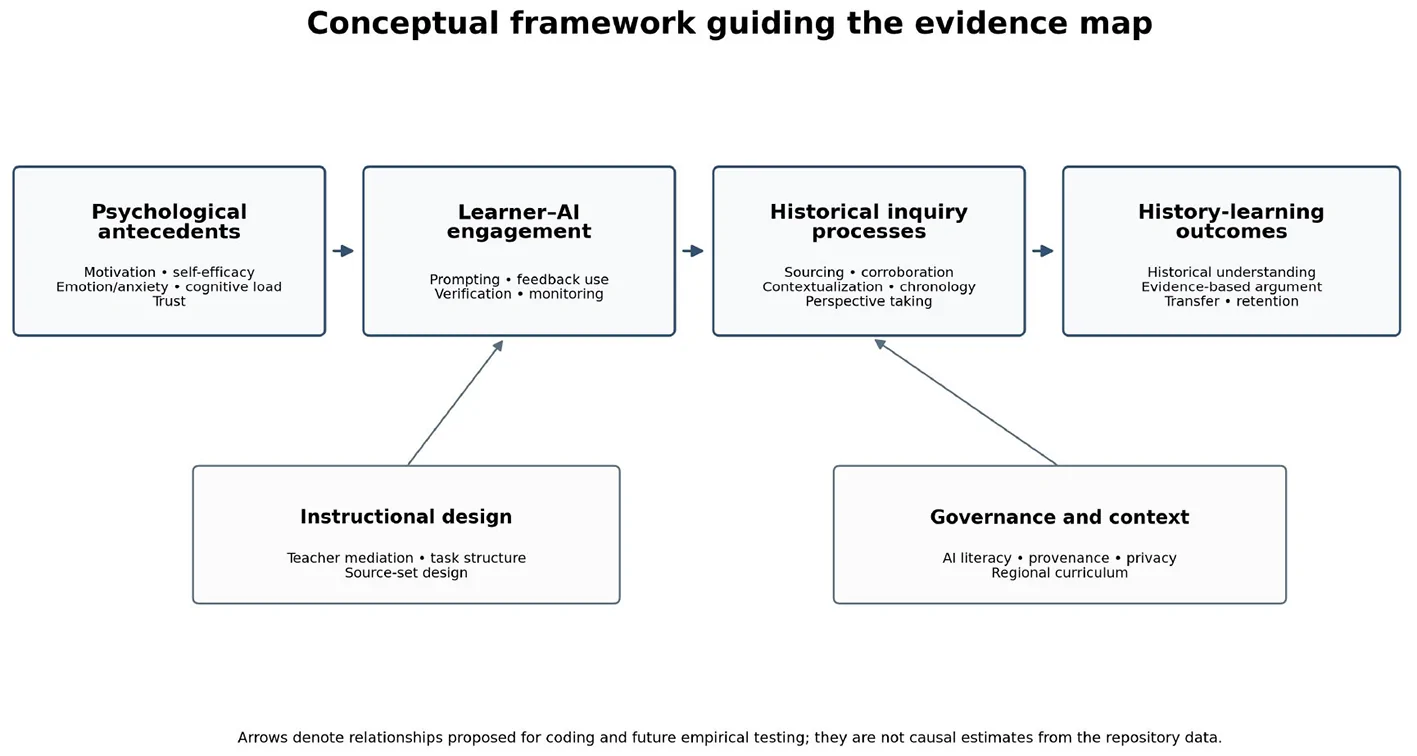
Conceptual framework guiding the evidence map. The arrows represent relationships proposed for coding and future empirical testing rather than causal effects established by the repository data.

## 5 Discussion

### 5.1 Principal findings

There are four analytic layers: a 30-record contextual repository map, nine records having an explicit mainland China, Hong Kong or Taiwan study context, five primary empirical studies from a subset of these, and a targeted full-text corroboration set of direct history-education evidence outside the repository. This layered structure is the principal methodological contribution of the study because it separates repository coverage from direct disciplinary evidence and prevents broader contextual studies from being misclassified as history-classroom evidence. The regional contextual evidence most frequently reflected emotion or cognitive load, engagement, and motivation. Their recurrence identifies candidate constructs for direct empirical testing; however, the regional frequencies do not indicate their relative importance in history classrooms. The directly relevant study on Guangzhou focuses on the change of paradigm from AI as an assistant to collaborative teacher-AI interaction, citing critical discussion, processing of historical data, and further exploration as examples (Li and Liao, 2025). This evidence is qualitative and is not meant to be combined with the regional frequency counts, but is to be read in conjunction with them.

The contextual outcome profile focused on immediate skills, knowledge, performance and problem solving. Additional sources identified by direct history-education studies and reviews include historical source selection, historical reasoning, perspective taking, and evidence-based interpretation, and the current evidence available for China is still not substantial enough for making comparative effectiveness claims. Delayed retention/transfer is a key priority for future work.

### 5.2 Theoretical interpretation

The proposed framework integrates motivational, self-regulatory, socio-technical, and historical-thinking approaches. It is designed for direct empirical testing: psychological conditions can affect learner-AI interaction; interaction can affect sourcing, corroboration, contextualization and monitoring; and these inquiry processes can help to shape historical understanding and evidence-based argument. These propositions are presented as a hypothesis-generating framework rather than as temporally ordered or empirically established causal relationships. The quality of motivation may be explained by self-determination theory (SDT). AI can help enhance autonomy in strategy selection, question posing and comparing options; competence in providing timely and comprehensible feedback; and relatedness in augmenting rather than supplanting human support in the classroom. But at the same time, this system can hinder the needs above by eliminating meaningful choice, making reasons invisible, or decreasing productive interaction (Ryan and Deci, 2020; Vansteenkiste et al., 2020). A second mechanism is through self-regulated learning.

Effective learners plan, monitor and reflect. These activities can be facilitated using AI, but AI can also do these activities for the learner. What counts here is retained agency (persons‘ goals, decisions, and verification remain with them). Darvishi et al. (2024) highlight the importance of student agency in AI-enhanced learning, and Chiu (2024) presents a framework for generative-AI support to students' self-regulation. While technology-acceptance constructs continue to serve as useful constructs for predicting user willingness to use AI, they should not be used as measures of learning. Adoption does not necessarily mean that the learners have acquired historical knowledge or historical thinking skills, but may be attributed to perceived usefulness and ease of use. Trust, too, needs tuning. Future history-education research could investigate whether learners validate information sources, acknowledge uncertainty, and question AI-generated interpretations when confronted with conflicting historical evidence. The modality of the AI is also important. While structured tutoring and robotics systems limit the number and length of tasks and feedback cycles, generative systems give the learner more freedom to make judgments, monitor, and verify the tasks. There is thus no single AI-to-performance model that will capture all forms of AI-assisted learning of history.

### 5.3 Evidence-based implications from direct and regional evidence

The practical conclusion is proposed with caution: AI is best suited in teacher-led inquiry rather than to replace the teacher's expertise in making pedagogical decisions for disciplinary studies (Li and Liao, 2025). The larger number of regional empirical studies also indicate that task structure, teacher support, engagement and learner confidence are important factors in implementation, but these studies were not conducted in history classrooms.

The obvious priority for history teaching in this regard, therefore, is to maintain teacher mediation and learner verification. History studies should mention whether students have compared the output produced by AI with primary and secondary sources, identified claims in the AI output that are not supported by the sources, described the changes made between drafts, and defended the interpretations they have made. These practices enable the making of historical reasoning visible and measurable outcomes for future assessment.

### 5.4 Hypothesis-generating implications for history education in China

The implications outlined below are ones of hypothesis generation and go beyond the very limited evidence base of direct links between history and education. AI literacy should be taught in the context of a particular historical discipline in relation to: Provenance of sources, fabricated citations, missing context, bias, uncertainty, authorship, privacy, and verification. To assess the plausibility and supporting evidence for an historical claim generated by AI, learners must also have enough domain knowledge (Ng et al., 2021; Chan, 2023; UNESCO, 2023; McGrew et al., 2018).

Differentiated support should be further tested in future studies as well. If a student's self-efficacy is low, it may be useful to give a structured source set and to have the student do the verification with scaffolding rather than on their own; if a student is anxious, it may be beneficial to give low-stakes practice before letting them choose their own sources to verify. For students who may be at greater risk of over-relying on AI, tasks such as source annotation, identification of counterevidence, and oral explanation can be used to preserve active learner reasoning. Moreover, empirical evidence from adult learning suggests that AI may be more beneficial when used to support reflection and self-directed learning rather than simple answer retrieval (Lin, 2024).

Regional contexts should not be assumed to be homogeneous. Mainland China, Hong Kong, and Taiwan differ in curriculum organization, policy environments, language practices, and patterns of educational technology use. Future studies should therefore report these settings separately and examine cost, access, privacy controls, platform language, and curriculum alignment.

### 5.5 Governance, ethics, and academic integrity

The policy considerations in this section are mainly from the general literature on AI in education and are suggested for assessment in the context of Chinese history education. Settings acceptable for AI usage can be identified by task type; disclosure should be required where appropriate; and there should be expectations regarding the provenance of the source, checking of citations, and independent reasoning (UNESCO, 2021, 2023; Cotton et al., 2024). Privacy is also in need of attention. Students may access personal, institutional or research data to external systems. Institutions can thus set forth clear rules, approved alternatives, and data retention, and model training risks information. The ethical guidance needs to be accessible to students and teachers, not limited to policy documents (UNESCO, 2021, 2023).

History teaching as a design issue is also a question of academic probity. Outsourcing generic essays is simple, but for those that require reasoning through local archives, annotated sources, staged drafts, oral defense, and explicit corroboration, reasoning becomes more visible. Assessment might then focus more on evidence use, evidence interpretation and clear reporting of support provided than on the quality of writing alone (Cotton et al., 2024). A layered governance model could be more suitable than general permission or prohibition: rules at the institutional level can cover privacy and approved tools, rules at the programme level can take into account the expectations of the discipline, and rules at the course level can capture disclosure, verification, and acceptable methods of assistance. These proposals will need to be assessed in Chinese history-education contexts before their effectiveness can be confirmed.

### 5.6 Research agenda

Future research should address questions that arise directly from the identified evidence gaps (Table 7). Priorities include controlled comparisons of ChatGPT- or large language model-assisted history teaching in mainland Chinese secondary schools, studies of sourcing and corroboration with and without AI assistance, and experiments comparing unguided AI use with teacher-scaffolded corroboration. Studies should precisely identify the type of AI system, the historical topic, the source set, the prompt and feedback structure, the teachers' role, the comparison condition and the type of work to be completed independently by the students. There is also work to be done longitudinally. Instruction should be followed up with delayed tests of historical knowledge, evaluation of sources, and transfer to unfamiliar documents. Contextual differences could be explored through parallel studies instead of being masked by the regional total if undertaken in mainland China, Hong Kong, and Taiwan.

**Table 7:** Research priorities linked directly to the identified evidence gaps.

Priority	Current gap	Recommended study feature
Direct mainland-China history evidence	Direct empirical evidence is sparse and absent from the original repository.	Run controlled ChatGPT/LLM-assisted history lessons in mainland Chinese secondary schools; measure sourcing, corroboration, historical argument, and delayed retention.
Source verification	The repository did not directly test historical source evaluation.	Compare sourcing and corroboration behavior with and without AI assistance using the same primary-source set.
Teacher mediation	Teacher support is repeatedly discussed but rarely isolated as a design factor.	Compare unguided AI use with teacher-scaffolded verification and source-checking protocols.
Psychological mechanisms	Emotion, anxiety, and cognitive load were grouped in first-pass coding.	Measure anxiety, cognitive load, motivation, self-efficacy, engagement, and trust separately with validated instruments.
Regional heterogeneity	Mainland China, Hong Kong, and Taiwan were previously summarized together.	Use parallel or stratified studies and report curriculum, policy, language, and platform context separately.
Chinese-language coverage	The original repository may underrepresent domestic literature and platforms.	Search CNKI, Wanfang, VIP, Scopus, Web of Science, and ERIC with equivalent Chinese/English terms and evaluate domestic Chinese-language AI tools.
Long-term learning	Delayed retention and transfer remain underexamined.	Add delayed post-tests and unfamiliar document-based transfer tasks after AI-assisted history units.

Psychological mechanisms, which were combined in the repository coding, should be separated in the measurement. Constructs such as motivation, self-efficacy, engagement, anxiety, cognitive load, trust, and agency should be assessed separately using validated instruments. History-learning outcomes should likewise be distinguished, including factual recall, sourcing, corroboration, contextualization, perspective taking, chronological reasoning, and evidence-based argument. In quantitative designs, use of significance tests should be accompanied by effect sizes and uncertainty.

Source coverage should be enhanced for China. In future searches, the key search words should be used in the same way as before, with the addition of CNKI, Wanfang Data, VIP, ERIC, Scopus and Web of Science as well as the equivalent Chinese and English terms; in empirical studies, the search should report the region, institution type, learner level, history topic, source language, platform, and curriculum context. Direct evaluation of Chinese-language and domestically developed AI systems should also be prioritized.

Qualitative work is still very important. Interviews, classroom observations, prompt logs, annotations, and source-use traces may reveal why students accept, reject, or challenge AI-generated historical interpretations. Mixed-method studies can then relate these interaction patterns to historical-thinking performance. Explanation tools are not assumed to build trust, but should be tried out with students (Khosravi et al., 2022).

### 5.7 Limitations

This evidence map has several important limitations. First, it is a secondary analysis of a single public repository export rather than a comprehensive multi-database systematic review. The export lacked candidate titles, publication years, journal names, document types, and DOI fields, and it was current only to 1 April 2024. Chinese-language databases, including CNKI, Wanfang Data, and VIP, were not independently searched; ERIC, Scopus, and Web of Science were also not searched *de novo*. The original repository may therefore omit Chinese-language studies, conference papers, non-English publications, and later work.

Second, regional classification was conservative. Nine records explicitly identified mainland China, Hong Kong, or Taiwan; 20 were location-unresolved; and one empirical study was explicitly located in South Korea. The South Korea record was retained only in the broad contextual map and excluded from regional interpretation. Mainland China, Hong Kong, and Taiwan also differ in curriculum, policy, language, and technology environments, so pooled regional frequencies should not be read as a homogeneous national estimate.

Third, full reports for the original candidate set were not available. Study design, sampling, instruments, analyses, attrition, intervention fidelity, and MMAT criteria could therefore not be verified consistently. Abstract-level evidence classifications remain provisional, and the MMAT readiness table documents missing information rather than methodological quality.

Fourth, repository coding relied on abstracts and keywords and was not independently double-coded. A second-pass audit was completed, but no inter-rater reliability statistic can be reported. Terminological variation may also have produced false negatives. The expanded synonym sensitivity audit reduced this concern but cannot exclude records that use unexpected discipline-specific language. The original combined category for emotion, anxiety, and cognitive load also limits psychological specificity.

Fifth, publication bias, selection bias, database bias, abstract-reporting bias, and methodological heterogeneity remain possible. The targeted full-text corroboration identified direct history-education evidence outside the original repository, demonstrating that the repository's empty direct-history result was a coverage limitation rather than a field-wide absence. Those external reports were not incorporated into repository denominators because they were not identified through the same search pathway.

The defensible contribution is therefore a transparent secondary evidence map, a clearly bounded regional subset, targeted corroboration of direct history-education evidence, and a set of testable propositions. A comprehensive update would require a *de novo* multi-database search, full report retrieval, independent double screening and coding, complete bibliographic identification, and full-text quality assessment.

## 6 Conclusion

The secondary evidence map shows that the original repository contains broad regional signals related to emotion or cognitive load, engagement, motivation, self-efficacy, skills, knowledge, performance, and problem solving, but it does not provide a comprehensive account of AI-assisted history education in China. Targeted full-text corroboration identified direct history-classroom evidence outside the repository, indicating that China-specific evidence is sparse rather than absent.

The strongest next step is direct testing of structured AI use, teacher mediation, learner agency, and verification in source-based history tasks. Outcomes should include sourcing, corroboration, contextualization, chronological reasoning, evidence-based argument, and delayed transfer, with regional settings reported separately.

For Chinese history education, AI use should be disciplined, transparent, and empirically evaluated rather than assumed to be beneficial. Learners should be taught to treat AI output as a claim requiring verification against historical evidence. Because the original export was current only to 1 April 2024 and later literature was considered separately, future work should use an updated multi-database search with Chinese-language coverage before broader conclusions are drawn.

## Data Availability

The original contributions presented in the study are included in the article/Supplementary material, further inquiries can be directed to the corresponding author.
