# Supplementary material for: AI-assisted history education in China: a secondary evidence map of psychological antecedents and learning outcomes
Source: Front Psychol. 2026 Sep 23;17:1956962. doi: 10.3389/fpsyg.2026.1956962 (PMC13642265; doi:10.3389/fpsyg.2026.1956962)
Supplement: Supplementary file 1 [file Supplementary_file_1.docx]

**Supplementary Material**

AI-Assisted History Education in China: A Secondary Evidence Map of Psychological Antecedents and Learning Outcomes

This package documents the secondary repository screening, record-level audit, reporting status, bibliographic checks, expanded terminology sensitivity audit, regional-classification rules, and targeted post-corpus full-text corroboration used in the manuscript. Repository-derived counts remain separate from externally supplied full-text reports.

# Supplementary Table S1. PRISMA 2020 / PRISMA-ScR reporting-status matrix

| **Item** | **Topic** | **Requirement** | **Location in manuscript** | **Status** |
| --- | --- | --- | --- | --- |
| 1 | Title | Identify the work accurately as a secondary evidence map rather than a comprehensive multi-database systematic review. | Title; Section 3.1 | Complete |
| 2 | Abstract | Report objectives, methods, results, and conclusions. | Abstract | Reported |
| 3 | Rationale | Explain the rationale in context. | Introduction | Reported |
| 4 | Objectives | State the objectives or questions. | Section 2.5 | Reported |
| 5 | Eligibility criteria | State inclusion and exclusion rules. | Section 3.3; Table 1 | Reported |
| 6 | Information sources | Identify all sources used. | Sections 3.1-3.2.1 | Complete for the repository and targeted full-text sources; no de novo database search |
| 7 | Search strategy | Provide complete database search strategies. | Sections 3.4 and 5.7; Table S11 | Complete for repository screening and sensitivity terms; not completed for a de novo multi-database search |
| 8 | Selection process | Describe selection and reviewer process. | Section 3.4 | Reported for repository screening and audit |
| 9 | Data collection | Describe how information was extracted. | Sections 3.4-3.7; Tables S2, S5, S6, S12 | Complete at abstract/metadata level for repository records; full-text extraction limited to targeted corroboration reports |
| 10a | Data items: outcomes | Define outcomes sought. | Sections 3.3 and 3.5 | Reported |
| 10b | Other variables | Define other variables sought. | Sections 3.3 and 3.5 | Reported |
| 11 | Risk of bias | Describe report-level quality appraisal. | Section 3.7; Table S3 | Not completed for the original candidate set because full reports were unavailable; treated as a central limitation |
| 12 | Effect measures | State effect measures. | Section 3.6 | Not applicable: no effect-size synthesis |
| 13a–f | Synthesis methods | Describe eligibility, preparation, tabulation, synthesis, heterogeneity, and sensitivity methods. | Sections 3.6-3.8 | Complete for descriptive evidence mapping and narrative synthesis; meta-analysis not applicable |
| 14 | Reporting bias assessment | Describe methods for reporting-bias assessment. | Section 5.6 | Discussed; no formal test possible |
| 15 | Certainty assessment | Describe certainty method. | Section 3.6 | Layered confidence interpretation reported; no formal GRADE-style certainty assessment |
| 16a–b | Study selection | Report selection results and exclusions. | Section 4.1; Figure 2; Tables S2 and S4 | Complete for repository screening and audit |
| 17 | Study characteristics | Cite each included report and present characteristics. | Table 3; Tables S5, S10, S12 | Partial for original repository records because some report-level fields were absent; targeted full-text sources are documented separately |
| 18 | Risk of bias in studies | Present quality-appraisal results. | Section 3.7; Table S3 | Not completed for original repository candidates |
| 19–22 | Results of studies and syntheses | Present individual and synthesized results, biases, and certainty. | Sections 4.2–4.6 | Descriptive evidence map only |
| 23a–d | Discussion | Interpret evidence, limitations, and implications. | Section 5 | Reported |
| 24a–c | Registration and protocol | Provide registration and protocol details. | Section 3.1 | Not registered |
| 25 | Support | Describe financial or non-financial support. | Declarations | No funding statement included in blinded file |
| 26 | Competing interests | Declare competing interests. | Blinded file | To be supplied by authors in journal system |
| 27 | Availability | State availability of data, code, and materials. | Data availability; this package | Reported |

# Supplementary Table S2. Candidate-record verification and decision log

| **EID** | **First author** | **Evidence type** | **Location status** | **Eligibility decision** | **Analysis role** |
| --- | --- | --- | --- | --- | --- |
| 2-s2.0-0025889819 | Ismail, Maizatul Akmar | Unclear/other | Confirmed mainland China/Hong Kong/Taiwan | Retain | Regional thematic map only |
| 2-s2.0-85049375612 | Flanagan, Brendan | Proceedings aggregate | Confirmed mainland China/Hong Kong/Taiwan | Retain | Regional thematic map only |
| 2-s2.0-85049379760 | Flanagan, Brendan | Proceedings aggregate | Confirmed mainland China/Hong Kong/Taiwan | Retain | Regional thematic map only |
| 2-s2.0-85089503148 | Ferrara, Lisa | Proceedings aggregate | Confirmed mainland China/Hong Kong/Taiwan | Retain | Regional thematic map only |
| 2-s2.0-85100559651 | Cross, Jeffrey S. | Empirical study | Location unresolved from supplied abstract | Retain as contextual evidence | Broad evidence map only |
| 2-s2.0-85110439050 | Chen, B.; Hwang, G.-H.; Wang, S.-H. | Empirical study | Confirmed mainland China/Hong Kong/Taiwan | Retain | Regional empirical analysis |
| 2-s2.0-85124236565 | Hwang, Gwo-Jen | Review/synthesis | Location unresolved | Exclude: learning outcome remained other/unclear. | Excluded from evidence map |
| 2-s2.0-85125240207 | Escamilla, Jose | Review/synthesis | Location unresolved from supplied abstract | Retain as contextual evidence | Broad evidence map only |
| 2-s2.0-85131220038 | Crompton, H. | Unclear/other | Location unresolved | Exclude: learning outcome remained other/unclear. | Excluded from evidence map |
| 2-s2.0-85132886754 | Chai, Ching Sing | Conceptual/system design | Location unresolved from supplied abstract | Retain as contextual evidence | Broad evidence map only |
| 2-s2.0-85132908819 | Ramiel, Hemy | Empirical study | Location unresolved from supplied abstract | Retain as contextual evidence | Broad evidence map only |
| 2-s2.0-85140063916 | du Boulay, Benedict | Empirical study | Confirmed non-regional study location: South Korea | Retain in broad contextual map; exclude from regional analysis | Contextual evidence only |
| 2-s2.0-85144925787 | Hu, Xiangen | Unclear/other | Location unresolved from supplied abstract | Retain as contextual evidence | Broad evidence map only |
| 2-s2.0-85146357857 | Elorriaga, Jon A. | Empirical study | Location unresolved from supplied abstract | Retain as contextual evidence | Broad evidence map only |
| 2-s2.0-85146954308 | Jeng, H.-L.; Chen, C.-N. | Empirical study | Confirmed mainland China/Hong Kong/Taiwan | Retain | Regional empirical analysis |
| 2-s2.0-85147012752 | Rego, Joilson | Empirical study | Location unresolved from supplied abstract | Retain as contextual evidence | Broad evidence map only |
| 2-s2.0-85147020204 | Ranchhod, Ashok | Empirical study | Location unresolved from supplied abstract | Retain as contextual evidence | Broad evidence map only |
| 2-s2.0-85147696412 | Leiker, Daniel | Empirical study | Location unresolved from supplied abstract | Retain as contextual evidence | Broad evidence map only |
| 2-s2.0-85147946187 | Do Nascimento, Felipe Barjud Pereira | Unclear/other | Location unresolved from supplied abstract | Retain as contextual evidence | Broad evidence map only |
| 2-s2.0-85160663282 | Adams, Catherine | Conceptual/system design | Location unresolved | Exclude: psychological antecedent remained other/unclear. | Excluded from evidence map |
| 2-s2.0-85165243775 | Kohnke, L.; Moorhouse, B. L.; Zou, D. | Empirical study | Confirmed mainland China/Hong Kong/Taiwan | Retain | Regional empirical analysis |
| 2-s2.0-85171282726 | Cheng, Eric C. K. | Empirical study | Location unresolved from supplied abstract | Retain as contextual evidence | Broad evidence map only |
| 2-s2.0-85173251462 | Crompton, H. | Empirical study | Location unresolved from supplied abstract | Retain as contextual evidence | Broad evidence map only |
| 2-s2.0-85177238794 | Fancsali, Stephen E. | Proceedings aggregate | Location unresolved | Exclude: learning outcome remained other/unclear. | Excluded from evidence map |
| 2-s2.0-85178289347 | Cheng, E. C. K.; Wang, T. | Empirical study | Confirmed mainland China/Hong Kong/Taiwan | Retain | Regional empirical analysis |
| 2-s2.0-85180332116 | Dyer, Joseph-Omer | Unclear/other | Location unresolved from supplied abstract | Retain as contextual evidence | Broad evidence map only |
| 2-s2.0-85180606431 | Sun, X.; Song, Y. | Empirical study | Confirmed mainland China/Hong Kong/Taiwan | Retain | Regional empirical analysis |
| 2-s2.0-85181168530 | Hu, Jingxyan | Review/synthesis | Location unresolved from supplied abstract | Retain as contextual evidence | Broad evidence map only |
| 2-s2.0-85181398793 | Raković, Mladen | Review/synthesis | Location unresolved from supplied abstract | Retain as contextual evidence | Broad evidence map only |
| 2-s2.0-85183815594 | Maphosa, Mfowabo | Unclear/other | Location unresolved from supplied abstract | Retain as contextual evidence | Broad evidence map only |
| 2-s2.0-85184994768 | Duan, Ye | Unclear/other | Location unresolved from supplied abstract | Retain as contextual evidence | Broad evidence map only |
| 2-s2.0-85185319165 | Reimann, Peter | Empirical study | Location unresolved from supplied abstract | Retain as contextual evidence | Broad evidence map only |
| 2-s2.0-85187799460 | Chen, Xieling | Conceptual/system design | Location unresolved | Exclude: learning outcome remained other/unclear. | Excluded from evidence map |
| 2-s2.0-85188926752 | Reimann, Peter | Empirical study | Location unresolved from supplied abstract | Retain as contextual evidence | Broad evidence map only |
| 2-s2.0-85188954746 | Dahri, Nisar Ahmed | Empirical study | Location unresolved from supplied abstract | Retain as contextual evidence | Broad evidence map only |

# Supplementary Table S3. MMAT 2018 readiness table for confirmed regional empirical studies

A definitive MMAT assessment was not completed for the original repository candidates because the full reports were not present. This table identifies information needed for later full-text appraisal and must not be interpreted as a quality score. Abstract-level study-design classifications remain provisional.

| **EID / author** | **Likely MMAT category** | **Screening question status** | **Design-specific criteria requiring full text** | **Current status** |
| --- | --- | --- | --- | --- |
| 2-s2.0-85110439050 / Chen, B.; Hwang, G.-H.; Wang, S.-H. | Quantitative non-randomized | Research question and relevant data visible in abstract | Representativeness; complete outcome data; measurement validity; confounders; intervention fidelity | Pending full report |
| 2-s2.0-85146954308 / Jeng, H.-L.; Chen, C.-N. | Mixed methods | Research questions and mixed data visible in abstract | Quality of qualitative and quantitative components; integration; divergence; component adherence | Pending full report |
| 2-s2.0-85165243775 / Kohnke, L.; Moorhouse, B. L.; Zou, D. | Qualitative | Aim and interview data visible in abstract | Appropriateness of approach; data collection; derivation; interpretation; coherence | Pending full report |
| 2-s2.0-85178289347 / Cheng, E. C. K.; Wang, T. | Qualitative | Aim and case-study data visible in abstract | Appropriateness of approach; sampling; data collection; analysis; coherence | Pending full report |
| 2-s2.0-85180606431 / Sun, X.; Song, Y. | Quantitative descriptive | Aim and survey data visible in abstract | Sampling relevance; representativeness; measurements; nonresponse; statistical appropriateness | Pending full report |

# Supplementary Table S4. Records excluded after construct/outcome eligibility audit

| **EID** | **First author** | **Reason for exclusion** |
| --- | --- | --- |
| 2-s2.0-85124236565 | Hwang, Gwo-Jen | Exclude: learning outcome remained other/unclear. |
| 2-s2.0-85131220038 | Crompton, H. | Exclude: learning outcome remained other/unclear. |
| 2-s2.0-85160663282 | Adams, Catherine | Exclude: psychological antecedent remained other/unclear. |
| 2-s2.0-85177238794 | Fancsali, Stephen E. | Exclude: learning outcome remained other/unclear. |
| 2-s2.0-85187799460 | Chen, Xieling | Exclude: learning outcome remained other/unclear. |

# Supplementary Table S5. Confirmed regional empirical study characteristics

| **EID / first author** | **Context/year signal** | **Sample** | **Design** | **System** | **Psychological construct** | **Outcome** | **Main abstract-level result** |
| --- | --- | --- | --- | --- | --- | --- | --- |
| 2-s2.0-85110439050 / Chen, B.; Hwang, G.-H.; Wang, S.-H. | Taiwan; 2021 copyright | 24 fourth-grade pupils | Classroom experiment | Game-based intelligent robot | Cognitive load; gender difference | Learning performance and efficiency | Boys showed lower cognitive load and higher efficiency; performance difference was not significant. |
| 2-s2.0-85146954308 / Jeng, H.-L.; Chen, C.-N. | Taiwan; 2022 copyright | 47 pupils aged 10–11 | Mixed methods | Digital Pentomino game and learning analytics | Expert–novice strategy differences | Spatial ability and problem-solving sequence | Experts outperformed novices and used different procedural strategies. |
| 2-s2.0-85165243775 / Kohnke, L.; Moorhouse, B. L.; Zou, D. | Hong Kong; 2023 copyright | 12 instructors | Qualitative interviews | Generative AI for language teaching | Attitude, intention, confidence | Readiness and anticipated learning support | Familiarity, confidence, institutional support, and professional development were prominent. |
| 2-s2.0-85178289347 / Cheng, E. C. K.; Wang, T. | Hong Kong; 2023 copyright | Not stated in abstract | Qualitative case study | Adaptive learning platform | Teacher engagement and implementation factors | Student engagement and learning-quality claims | Leadership support and student-centred pedagogy were highlighted. |
| 2-s2.0-85180606431 / Sun, X.; Song, Y. | China; 2023 copyright | 750 teachers | Cross-sectional survey | AI and big-data analytics | Technological literacy | Teacher performance | Positive associations were reported; the design does not establish causality. |

# Supplementary Table S6. Retained broad evidence-map coding

| **EID** | **First author** | **Location status** | **Evidence type** | **Sector** | **AI modality** | **Psychological codes** | **Outcome codes** |
| --- | --- | --- | --- | --- | --- | --- | --- |
| 2-s2.0-0025889819 | Ismail, Maizatul Akmar | Confirmed region | Unclear/other | Higher education | Adaptive/ITS | Motivation/interest | Knowledge/understanding; Skills/competence; Problem solving/critical thinking |
| 2-s2.0-85049375612 | Flanagan, Brendan | Confirmed region | Proceedings aggregate | Vocational | Generative AI/chatbot; Adaptive/ITS; Educational robotics | Emotion/anxiety/cognitive load; Engagement; Self-efficacy/competence | Knowledge/understanding; Skills/competence; Problem solving/critical thinking |
| 2-s2.0-85049379760 | Flanagan, Brendan | Confirmed region | Proceedings aggregate | Vocational | Generative AI/chatbot; Adaptive/ITS; Educational robotics | Emotion/anxiety/cognitive load; Engagement; Self-efficacy/competence | Knowledge/understanding; Skills/competence; Problem solving/critical thinking |
| 2-s2.0-85089503148 | Ferrara, Lisa | Confirmed region | Proceedings aggregate | Mixed/not stated | Adaptive/ITS; Analytics/prediction | Emotion/anxiety/cognitive load; Acceptance/attitude/intention; Self-regulation/agency | Achievement/performance |
| 2-s2.0-85100559651 | Cross, Jeffrey S. | Location unresolved | Empirical study | Higher education | General AI | Motivation/interest | Knowledge/understanding |
| 2-s2.0-85110439050 | Chen, B.; Hwang, G.-H.; Wang, S.-H. | Confirmed region | Empirical study | K-12 | Educational robotics | Emotion/anxiety/cognitive load | Achievement/performance; Efficiency/productivity |
| 2-s2.0-85125240207 | Escamilla, Jose | Location unresolved | Review/synthesis | Higher education | Analytics/prediction; Automated feedback/assessment | Engagement; Satisfaction/enjoyment | Achievement/performance; Satisfaction |
| 2-s2.0-85132886754 | Chai, Ching Sing | Location unresolved | Conceptual/system design | Mixed/not stated | Adaptive/ITS; Analytics/prediction | Trust | Knowledge/understanding; Skills/competence |
| 2-s2.0-85132908819 | Ramiel, Hemy | Location unresolved | Empirical study | K-12 | General AI | Motivation/interest; Emotion/anxiety/cognitive load | Achievement/performance; Skills/competence; Problem solving/critical thinking |
| 2-s2.0-85144925787 | Hu, Xiangen | Location unresolved | Unclear/other | Higher education | Generative AI/chatbot; Analytics/prediction | Emotion/anxiety/cognitive load; Self-efficacy/competence; Self-regulation/agency; Satisfaction/enjoyment | Achievement/performance; Knowledge/understanding; Skills/competence; Satisfaction |
| 2-s2.0-85146357857 | Elorriaga, Jon A. | Location unresolved | Empirical study | Higher education | Analytics/prediction | Engagement; Satisfaction/enjoyment | Achievement/performance; Satisfaction |
| 2-s2.0-85146954308 | Jeng, H.-L.; Chen, C.-N. | Confirmed region | Empirical study | Higher education | Analytics/prediction | Motivation/interest | Achievement/performance; Skills/competence; Problem solving/critical thinking; Efficiency/productivity |
| 2-s2.0-85147012752 | Rego, Joilson | Location unresolved | Empirical study | Higher education | Analytics/prediction | Motivation/interest | Achievement/performance |
| 2-s2.0-85147020204 | Ranchhod, Ashok | Location unresolved | Empirical study | Mixed/not stated | Generative AI/chatbot | Emotion/anxiety/cognitive load; Satisfaction/enjoyment | Achievement/performance; Knowledge/understanding |
| 2-s2.0-85147696412 | Leiker, Daniel | Location unresolved | Empirical study | K-12 | Generative AI/chatbot | Motivation/interest; Self-efficacy/competence; Self-regulation/agency; Satisfaction/enjoyment | Achievement/performance; Knowledge/understanding; Skills/competence; Satisfaction |
| 2-s2.0-85147946187 | Do Nascimento, Felipe Barjud Pereira | Location unresolved | Unclear/other | Mixed/not stated | General AI | Emotion/anxiety/cognitive load | Skills/competence |
| 2-s2.0-85165243775 | Kohnke, L.; Moorhouse, B. L.; Zou, D. | Confirmed region | Empirical study | Higher education | Generative AI/chatbot | Self-efficacy/competence; Acceptance/attitude/intention | Knowledge/understanding; Skills/competence |
| 2-s2.0-85171282726 | Cheng, Eric C. K. | Location unresolved | Empirical study | K-12 | Educational robotics | Motivation/interest; Self-efficacy/competence | Knowledge/understanding; Skills/competence |
| 2-s2.0-85173251462 | Crompton, H. | Location unresolved | Empirical study | Mixed/not stated | Analytics/prediction | Engagement; Satisfaction/enjoyment | Achievement/performance; Skills/competence; Satisfaction |
| 2-s2.0-85178289347 | Cheng, E. C. K.; Wang, T. | Confirmed region | Empirical study | K-12 | Adaptive/ITS | Motivation/interest; Engagement | Achievement/performance; Knowledge/understanding |
| 2-s2.0-85180332116 | Dyer, Joseph-Omer | Location unresolved | Unclear/other | Vocational | General AI | Motivation/interest | Achievement/performance; Problem solving/critical thinking; Creativity |
| 2-s2.0-85180606431 | Sun, X.; Song, Y. | Confirmed region | Empirical study | Higher education | General AI | Emotion/anxiety/cognitive load; Engagement | Achievement/performance; Skills/competence |
| 2-s2.0-85181168530 | Hu, Jingxyan | Location unresolved | Review/synthesis | Higher education | Generative AI/chatbot | Self-efficacy/competence; Acceptance/attitude/intention | Skills/competence |
| 2-s2.0-85181398793 | Raković, Mladen | Location unresolved | Review/synthesis | Mixed/not stated | Generative AI/chatbot | Emotion/anxiety/cognitive load | Knowledge/understanding; Efficiency/productivity |
| 2-s2.0-85183815594 | Maphosa, Mfowabo | Location unresolved | Unclear/other | Higher education | Generative AI/chatbot; Adaptive/ITS; Analytics/prediction | Engagement | Knowledge/understanding; Skills/competence; Problem solving/critical thinking; Creativity |
| 2-s2.0-85184994768 | Duan, Ye | Location unresolved | Unclear/other | Mixed/not stated | Adaptive/ITS | Motivation/interest; Engagement | Achievement/performance; Knowledge/understanding; Efficiency/productivity |
| 2-s2.0-85185319165 | Reimann, Peter | Location unresolved | Empirical study | Higher education | Generative AI/chatbot | Satisfaction/enjoyment | Knowledge/understanding; Problem solving/critical thinking; Efficiency/productivity; Satisfaction |
| 2-s2.0-85188926752 | Reimann, Peter | Location unresolved | Empirical study | Higher education | General AI | Motivation/interest; Emotion/anxiety/cognitive load; Self-efficacy/competence | Knowledge/understanding; Skills/competence; Problem solving/critical thinking |
| 2-s2.0-85188954746 | Dahri, Nisar Ahmed | Location unresolved | Empirical study | Higher education | General AI | Emotion/anxiety/cognitive load | Efficiency/productivity |
| 2-s2.0-85140063916 | du Boulay, Benedict | South Korea; excluded from regional counts | Empirical study | Higher education | General AI | Emotion/anxiety/cognitive load; Acceptance/attitude/intention | Achievement/performance; Knowledge/understanding; Skills/competence; Creativity |

# Supplementary Table S7. Screening dictionary and post-screening rules

| **Domain** | **Illustrative terms or rules** |
| --- | --- |
| AI in education | artificial intelligence; intelligent tutor; adaptive learning; chatbot; generative AI; learning analytics; educational robot; automated feedback |
| China signal | China; Chinese; Hong Kong; Taiwan; mainland; or affiliation signal during first-stage screening |
| Psychological construct | motivation; interest; emotion; anxiety; cognitive load; engagement; self-efficacy; confidence; competence; attitude; acceptance; intention; trust; satisfaction; autonomy; agency; self-regulation |
| Learning outcome | achievement; performance; knowledge; understanding; skill; competence; problem solving; critical thinking; efficiency; satisfaction; creativity; retention; persistence |
| Post-screening location rule | Affiliation alone did not establish study location. Regional analysis required an explicit mainland China, Hong Kong, or Taiwan study signal. |
| Post-screening eligibility rule | Both an identifiable psychological construct and an identifiable educational outcome were required. Other/unclear alone was insufficient. |
| Evidence-type rule | Primary empirical records supported observed-result interpretation. Other record types supported topic mapping only. |
| Expanded AI sensitivity terms | artificial intelligence; AI; machine learning; deep learning; large language model; LLM; generative AI; ChatGPT; chatbot; intelligent tutoring; learning analytics; educational robotics; adaptive learning; natural language processing |
| Expanded history sensitivity terms | history education; history teaching; history learning; historical thinking; historical reasoning; historical inquiry; historical literacy; source evaluation; sourcing; corroboration; contextualization; chronology; social studies; humanities; Chinese history; world history |

# Supplementary Table S8. DOI and reference-list matching audit

| **DOI** | **First author/source** | **Year** | **Reference title** | **Internal status** |
| --- | --- | --- | --- | --- |
| 10.1016/j.heliyon.2024.e31887 | Almogren | 2024 | Exploring factors influencing the acceptance of ChatGPT in higher education: A smart education perspective | Matched to manuscript reference list |
| 10.1007/s10639-023-12223-4 | Ansari | 2024 | Mapping the global evidence around the use of ChatGPT in higher education: A systematic scoping review | Matched to manuscript reference list |
| 10.1186/s41239-023-00408-3 | Chan | 2023 | A comprehensive AI policy education framework for university teaching and learning | Matched to manuscript reference list |
| 10.1186/s41239-023-00411-8 | Chan | 2023 | Students’ voices on generative AI: Perceptions, benefits, and challenges in higher education | Matched to manuscript reference list |
| 10.1109/ACCESS.2020.2988510 | Chen | 2020 | Artificial intelligence in education: A review | Matched to manuscript reference list |
| 10.1016/j.chb.2021.106909 | Chiu | 2021 | Digital support for student engagement in blended learning based on self-determination theory | Matched to manuscript reference list |
| 10.1080/15391523.2021.1891998 | Chiu | 2022 | Applying the self-determination theory to explain student engagement in online learning during the COVID-19 pandemic | Matched to manuscript reference list |
| 10.1007/s11423-024-10366-w | Chiu | 2024a | A classification tool to foster self-regulated learning with generative artificial intelligence by applying self-determination theory: A case of ChatGPT | Matched to manuscript reference list |
| 10.1016/j.caeai.2023.100197 | Chiu | 2024b | Future research recommendations for transforming higher education with generative AI | Matched to manuscript reference list |
| 10.1080/10494820.2023.2253861 | Chiu | 2024c | The impact of generative AI on practices, policies and research direction in education: A case of ChatGPT and Midjourney | Matched to manuscript reference list |
| 10.1016/j.caeai.2022.100118 | Chiu | 2023 | Systematic literature review on opportunities, challenges, and future research recommendations of artificial intelligence in education | Matched to manuscript reference list |
| 10.1080/14703297.2023.2190148 | Cotton | 2024 | Chatting and cheating: Ensuring academic integrity in the era of ChatGPT | Matched to manuscript reference list |
| 10.1186/s41239-023-00392-8 | Crompton | 2023 | Artificial intelligence in higher education: The state of the field | Matched to manuscript reference list |
| 10.1016/j.compedu.2023.104967 | Darvishi | 2024 | Impact of AI assistance on student agency | Matched to manuscript reference list |
| 10.1186/s41239-024-00452-7 | Delcker | 2024 | First-year students’ AI-competence as a predictor for intended and de facto use of AI-tools for supporting learning processes in higher education | Matched to manuscript reference list |
| 10.3389/feduc.2023.1206936 | Dempere | 2023 | The impact of ChatGPT on higher education | Matched to manuscript reference list |
| 10.1016/j.compedu.2024.105224 | Deng | 2025 | Does ChatGPT enhance student learning? A systematic review and meta-analysis of experimental studies | Matched to manuscript reference list |
| 10.1186/s41239-023-00434-1 | Ding | 2023 | Students’ perceptions of using ChatGPT in a physics class as a virtual tutor | Matched to manuscript reference list |
| 10.1186/s41239-023-00425-2 | Escalante | 2023 | AI-generated feedback on writing: Insights into efficacy and ENL student preference | Matched to manuscript reference list |
| 10.1016/j.caeai.2023.100198 | Essel | 2024 | ChatGPT effects on cognitive skills of undergraduate students: Receiving instant responses from AI-based conversational large language models | Matched to manuscript reference list |
| 10.1038/s41598-025-06930-w | Fan | 2025 | Educational impacts of generative artificial intelligence on learning and performance of engineering students in China | Matched to manuscript reference list |
| 10.1080/14703297.2023.2195846 | Farrokhnia | 2024 | A SWOT analysis of ChatGPT: Implications for educational practice and research | Matched to manuscript reference list |
| 10.3390/educsci13070692 | Grassini | 2023 | Shaping the future of education: Exploring the potential and consequences of AI and ChatGPT in educational settings | Matched to manuscript reference list |
| 10.1177/08295735211055355 | Guay | 2022 | Applying self-determination theory to education: Regulation types, psychological needs, and autonomy-supporting behaviors | Matched to manuscript reference list |
| 10.3233/EFI-180221 | Hong | 2018 | The Mixed Methods Appraisal Tool (MMAT) version 2018 for information professionals and researchers | Matched to manuscript reference list |
| 10.1016/j.caeai.2020.100001 | Hwang | 2020 | Vision, challenges, roles and research issues of artificial intelligence in education | Matched to manuscript reference list |
| 10.30935/cedtech/13605 | Imran | 2023 | Analyzing the role of ChatGPT as a writing assistant at higher education level: A systematic review of the literature | Matched to manuscript reference list |
| 10.3390/bs15101348 | Jia | 2025 | Effect of GenAI dependency on university students’ academic achievement: The mediating role of self-efficacy and moderating role of perceived teacher caring | Matched to manuscript reference list |
| 10.1016/j.lindif.2023.102274 | Kasneci | 2023 | ChatGPT for good? On opportunities and challenges of large language models for education | Matched to manuscript reference list |
| 10.1016/j.caeai.2022.100074 | Khosravi | 2022 | Explainable artificial intelligence in education | Matched to manuscript reference list |
| 10.1177/10451595231184928 | Lin | 2024 | Exploring the role of ChatGPT as a facilitator for motivating self-directed learning among adult learners | Matched to manuscript reference list |
| 10.1186/s41239-025-00567-5 | Liu | 2025 | Assessing strategic use of artificial intelligence in self-regulated learning: Instrument development and evidence from Chinese university students | Matched to manuscript reference list |
| 10.3390/educsci13040410 | Lo | 2023 | What is the impact of ChatGPT on education? A rapid review of the literature | Matched to manuscript reference list |
| 10.1016/j.compedu.2024.105100 | Lo | 2024 | The influence of ChatGPT on student engagement: A systematic review and future research agenda | Matched to manuscript reference list |
| 10.17632/46xbj75f6n.1 | Hanid, F. | 2025 | Bibliometric Dataset on AI in Education (Version 1) [Data set]. Mendeley Data. | Matched to manuscript reference list |
| 10.1016/j.caeai.2021.100041 | Ng | 2021 | Conceptualizing AI literacy: An exploratory review | Matched to manuscript reference list |
| 10.1016/j.caeai.2021.100020 | Ouyang | 2021 | Artificial intelligence in education: The three paradigms | Matched to manuscript reference list |
| 10.1007/s10639-022-10925-9 | Ouyang | 2022 | Artificial intelligence in online higher education: A systematic review of empirical research from 2011 to 2020 | Matched to manuscript reference list |
| 10.1136/bmj.n71 | Page | 2021 | The PRISMA 2020 statement: An updated guideline for reporting systematic reviews | Matched to manuscript reference list |
| 10.3390/app13095783 | Rahman | 2023 | ChatGPT for education and research: Opportunities, threats, and strategies | Matched to manuscript reference list |
| 10.1007/s40593-016-0110-3 | Roll | 2016 | Evolution and revolution in artificial intelligence in education | Matched to manuscript reference list |
| 10.1016/j.cedpsych.2020.101860 | Ryan | 2020 | Intrinsic and extrinsic motivation from a self-determination theory perspective: Definitions, theory, practices, and future directions | Matched to manuscript reference list |
| 10.1186/s40561-023-00237-x | Tlili | 2023 | What if the devil is my guardian angel: ChatGPT as a case study of using chatbots in education | Matched to manuscript reference list |
| 10.1007/s11031-019-09818-1 | Vansteenkiste | 2020 | Basic psychological need theory: Advancements, critical themes, and future directions | Matched to manuscript reference list |
| 10.1186/s41239-019-0171-0 | Zawacki-Richter | 2019 | Systematic review of research on artificial intelligence applications in higher education: Where are the educators? International Journal of Educational Technology in Higher Education, 16, 39 | Matched to manuscript reference list |
| 10.1155/2021/8812542 | Zhai | 2021 | A review of artificial intelligence in education from 2010 to 2020 | Matched to manuscript reference list |

The audit confirms internal matching and identifier formatting within the supplied manuscript. The Roll and Wylie (2016) DOI has been corrected to 10.1007/s40593-016-0110-3. Publisher landing-page validation was not available in the supplied archive.

Supplementary Table S9. History-domain audit and broadened sensitivity check

| Audit domain | Search terms or rule | Result |
| --- | --- | --- |
| Direct history-education terms | history education; history teaching; history learning; history classroom; history curriculum | No directly eligible history-education record identified in the original repository |
| Historical-thinking terms | historical thinking; historical reasoning; sourcing; corroboration; contextualization; chronological reasoning | No directly eligible history-education record identified in the original repository |
| Source-based inquiry terms | document-based inquiry; historical sources; historiography; evidence-based historical argument | No directly eligible history-education record identified in the original repository |
| Interpretive rule | A direct history-domain term had to appear in the abstract or keywords. | Broader social-studies/humanities matches required manual history-domain confirmation |
| Expanded AI dictionary | AI and related terms listed in Table S7 | AI-screen count remained n = 1,026 |
| Expanded history-adjacent dictionary | social studies; humanities; learning history; sourcing; contextualization and related terms | 13 records matched at least one broadened term; manual inspection found no direct history-education study |

Supplementary Table S10. Bibliographic details and stable identifiers for the nine regional records

Note. The original nine regional records retain EIDs where report-level fields were unavailable in the seven-field export. These identifiers remain auditable but are not treated as substitutes for full bibliographic retrieval. Direct history-education reports supplied outside the repository are documented separately in Table S12.

Supplementary Table S11. Expanded terminology sensitivity audit

| Audit component | Terms added or broadened | Repository result | Interpretation |
| --- | --- | --- | --- |
| AI terminology | machine learning; deep learning; large language model; LLM; generative AI; ChatGPT; chatbot; intelligent tutoring; learning analytics; educational robotics; adaptive learning; natural language processing | n = 1,026 AI-screen records, unchanged from the primary screen | The original AI screen was not sensitive to the omission of these common AI synonyms. |
| History-domain sensitivity | historical inquiry; historical literacy; source evaluation; sourcing; corroboration; contextualization; chronology; historical empathy; social studies; humanities; Chinese history; world history; learning history | 13 repository records matched at least one broadened history-adjacent term | Manual inspection did not identify a direct history-education study; the repository result remained empty for direct history evidence. |
| Interpretive boundary | social studies and humanities | Manual verification required | Broad disciplinary labels were not counted as history education without an explicit history-specific context. |

Supplementary Table S12. Targeted post-corpus full-text corroboration

| Source | Year | Evidence type | Setting / scope | Directness to Chinese history education | Key contribution used in manuscript | Identifier |
| --- | --- | --- | --- | --- | --- | --- |
| Li, Y.; Liao, N. | 2025 | Primary qualitative / grounded-theory study | 36 history teachers from eight secondary schools in Guangzhou, China | Direct | Documents AI-assisted teaching models in secondary-school history classrooms and the shift from replacement toward teacher-AI collaboration. | https://doi.org/10.70695/IAA1202501A10 |
| Mahmudi, A. R.; Manurung, N. T.; Ibrahim, N.; Suryadi, A. | 2026 | Systematic literature review | AI in history learning; database search across Scopus, Web of Science, and ERIC | Direct to history education; not China-specific | Confirms a broader direct history-learning literature and provides an external benchmark for search coverage. | https://doi.org/10.29408/fhs.v10i2.34717 |
| Li, H.; Wang, J.; Zhu, Z.; Wang, H. | n.d. | Systematic literature review manuscript | 18 empirical studies in AI and history education | Direct to history education; mixed international scope | Provides a second independent full-text benchmark and a seed bibliography for report-level retrieval. | Publication details not shown in the supplied manuscript |
| Yu, Y. | 2026 | Primary mixed-method empirical study | AI-assisted contextual narratives for Chinese cultural-heritage education | Adjacent, not direct school-history evidence | Provides China-focused evidence on knowledge retention, engagement, cultural identity, and AI-assisted historical/cultural contextualization. | https://doi.org/10.1145/3828733 |

Supplementary Table S13. Regional interpretation rules

| Regional status | Analytic treatment | Reason |
| --- | --- | --- |
| Mainland China | Reported as the primary national context where study location is explicit. | Directly matches the manuscript focus. |
| Hong Kong | Reported separately within the regional evidence layer. | Curriculum, policy, and language context may differ from mainland China. |
| Taiwan | Reported separately within the regional evidence layer. | Curriculum, policy, and language context may differ from mainland China. |
| Location unresolved | Retained only in the broad contextual map. | Affiliation or abstract text is insufficient to establish data-collection location. |
| South Korea | Retained in the broad contextual map but excluded from regional interpretation. | The study location is explicitly outside the regional focus. |
